# Food and housing security at a US Hispanic-Serving Institution: An examination before and during the COVID-19 pandemic

**DOI:** 10.3389/fpubh.2022.918955

**Published:** 2022-07-27

**Authors:** Amy Wagler, Gregory S. Schober, Silvia M. Chavez-Baray, Jessica Ayala, Paul R. Dessauer, Eva M. Moya

**Affiliations:** ^1^Department of Mathematical Sciences, College of Science, University of Texas at El Paso, El Paso, TX, United States; ^2^Department of Rehabilitation Sciences, College of Health Sciences, University of Texas at El Paso, El Paso, TX, United States; ^3^Department of Social Work, College of Health Sciences, University of Texas at El Paso, El Paso, TX, United States; ^4^Chicano Studies, College of Liberal Arts, University of Texas at El Paso, El Paso, TX, United States

**Keywords:** Hispanic-Serving University, border, food insecurity, COVID-19, health

## Abstract

University students occupy a socially marginal position and therefore are often underserved by academic and service institutions. This article analyzes food and housing security among students at The University of Texas at El Paso, a Hispanic-Serving Institution located in the U.S.-Mexico Border region. Findings of a sample of *n* = 7,633 university students are presented in the first cross-sectional, two-year food and housing security study on campus administered *via* platform Campus Labs Baseline. The first sample in 2019 consisted of *n* = 2,615 students representing 10.4% of student enrollment (25,177 total 2019 enrollment), and the second sample in 2020 was *n* = 5,018 representing 20.2% of student enrollment (24,879 total 2020 enrollment). To measure food security, the six-item short form of the U.S. Department of Agriculture (USDA) Household Food Security Survey Module was used. To document housing security, we created questions informed by student input. In this study, survey results are reported, and tests are conducted to assess the relationships between various student characteristics and food and housing security. Student characteristics significantly impacting food and housing security are probed further using data visualizations and subpopulation analysis with a focus on analyzing factors impacted by the COVID-19 pandemic. Results indicate that employment status, consistent employment status, hours per week, academic level, number of dependents, and gender are all factors associated with food security during the pandemic but not prior to the pandemic. Other factors, including, college affiliation, ethnicity/race, having any dependents and being head of household, living alone, mode of campus transportation and mode of the transportation, household income, and age, all were associated with food security in both academic years. Using these results, a critical analysis of past interventions addressing food and housing security is presented with a focus on changes made during the pandemic. Recommendations are made for further data-driven interventions and future steps.

## Introduction

Public health and health equality are essential for human development. Health is both a medical and social issue compounded by structural, economic, and environmental factors. If these factors are compromised, vulnerabilities can create health inequalities and human disasters (1). Low socioeconomic status is associated with poor birth outcomes, infectious diseases, chronic conditions, and life expectancy, which result from disparities that include poor access to health care, financial constraints, environmental differences, differential access to information, geographic locality, and behavioral factors (2). Economic instability is associated with worse health outcomes, forcing individuals to prioritize other issues such as rent and utility bills over food and health needs. Some key barriers to obtaining food include reduced access to supermarkets with healthier food options, as well as difficulty accessing federal nutrition assistance programs and food from food banks or pantries due to lack of these nearby, lack of transportation to get to them and complicated and time-consuming application process to access federal assistance. Informational barriers like the lack of awareness or understanding about available food and housing resources also may contribute to low utilization. In addition, the stigma associated with participation in public assistance programs may affect access as well (3).

Food security (FS) is “access by all people at all times to enough food for an active, healthy life” (4). Food insecurity “exists whenever the availability of nutritionally adequate and safe foods or the ability to acquire acceptable foods in socially acceptable ways is limited or uncertain” [(5), p. 1560]. Food insecurity is a risk factor for all types of malnutrition, food deficiencies, excess or imbalance of energy, as well as under and over nutrition like being overweight or obese due to insufficient intake and overconsumption of high-calorie/low-nutrient-dense foods (6). Food insecurity is more prevalent in urban areas, immigrant communities and among racial/ethnic groups, which are tied to lack to equity of resources leading to poor health outcomes that during periods of economic downturn, tend to increase (7). In addition, systemic inequities drive food and nutrition insecurity. Differences between racial and ethnic groups highlight a lack of equity that may lead to health disparities among food-insecure populations (8).

Housing security (HS) is defined as “availability of and access to stable, safe, adequate, and affordable housing and neighborhoods regardless of gender, race, ethnicity, or sexual orientation” [(9), p. 99]. Housing insecurity is a lack of access to safe, affordable, and quality housing, and it includes homelessness, housing instability, poor housing conditions, and low household or neighborhood safety (9). Housing insecurity is a determinant of multiple high-risk behaviors and poor health outcomes among adults (10), and it also contributes to several low health outcomes among children (11). In the United States, approximately one in 10 college students is homeless and 45% live in an unsafe environment with a wide range of challenges related to housing affordability and stability (12).

The relationship between education and health at both individual and regional levels is salient (1). In the United States, accessibility to colleges and universities has increased in the past 50 years, resulting in demographic composition changes with more low-income, first-generation, racial, and ethnic minority students enrolled than ever before (13, 14).

Nationally, the demographic characteristics of university students are shifting, and it is becoming more common for students to have children and work full-time while enrolled as full-time students (14). Food insecure students are also more likely than food secure students to experience housing insecurity, gain weight while attending college, partake in unhealthy diets with higher sugar and fat content, and experience psychological distress (15).

Among higher education students, basic needs insecurity—which includes food and housing insecurity—contributes to poor academic and health outcomes. Food and housing security is a basic need and if students' needs are not met, then they will be unable to engage in higher-level learning (13). Basic needs insecurity among college and university students is associated with several negative health outcomes, including decreased cognition and sleep quality, increased rates of certain chronic diseases, higher body mass index, higher odds of stress and depression, more emergency room visits and hospitalizations, and higher mortality rates (7, 13, 14).

A study by College and University Food Bank Alliance (16–18) revealed that 30% of college students in the U.S. are food insecure, and 56% of these students are employed, 75% receive financial aid and 43% participate in some type of campus meal plan. In addition, 36% are housing insecure, a number that increases to 51% for community college students, and 14% of students are homeless. The growing cost of campus tuition, health care, books, transportation, and living expenses have resulted in students having to decide between paying for bills or securing food forcing some students to leave college without obtaining degrees with financial concerns as the primary cause (16–18).

The COVID-19 pandemic exacerbated the financial challenges for many US households. Higher unemployment due to lockdowns and social distancing measures resulted in new or worsening economic barriers to basic needs security. In addition, public transportation was disrupted due to social distancing requirements, presenting a physical barrier to obtaining food for millions of Americans (7).

While young people are less vulnerable to severe illness from COVID-19, their education, work, and social lives have been interrupted by the pandemic (19). These interruptions have important consequences for public health, including an increase in anxiety and depressive symptoms and increased risk of psychiatric diagnosis (20). Beyond mental health, the combination of COVID-19 and food insecurity was found to promote gut anomalies, which could have acute or long-term health implications for infections and chronic conditions (21).

### Importance of university response to FS and HS

It is critical to improve our understanding of the impact of the COVID-19 pandemic on food and housing security among higher education students. By measuring changes in basic needs security for this population, we can prepare for the likely public health and social consequences in the short and medium term. Furthermore, by identifying the key factors that are associated with food and housing security, we can more effectively direct limited resources to the students who are most in need and improve student academic outcomes in the long run. In this article, we analyze FS and HS among higher education students. The paper focuses on variables of importance that contribute to food and housing security to highlight some of the differences that coincided with the COVID-19 pandemic. In conclusion, we make recommendations for other institutions experiencing similar effects of the pandemic on student food and housing security.

## Materials and methods

### Participants

The study used a cross-sectional, survey-based design to examine FS and HS among university students at an urban Hispanic-Serving Institution. The survey study compares 2 years of data, including before and during the COVID-19 pandemic. The study setting is a Hispanic-Serving University located in the U.S.-Mexico border region. The student population is representative of the local community: over 83% of students are Hispanic and nearly 50% self-identify as a first-generation student (22).

### Procedure

In 2019 and 2020, online surveys were administered to students *via* a university platform to collect, analyze, and translate data in real time. Author and co-authors prepared the study protocol and instrument, which was piloted in the focused population by a trained interviewer (first and senior author), and student feedback from the pilot survey helped inform the final version of the survey questions. Using a Customer Relationship Management Program (CRM), survey invitations were sent to all students at the HSI in Fall 2019 (October 7–23, 2019) and Fall 2020 (November 5–20, 2020). The student population over the age of 18 enrolled at the university in 2019 was 25,177, and in 2020 was 24,879. Four emails were sent by CRM, including the initial invitation and three reminders in both years. Participants who voluntarily accepted to be in the study consented electronically and completed the survey online. The survey contained 30–36 questions, took approximately 10 min to complete, was anonymous, and was open for at least 16 days each year. Participants had the option to enter a raffle for four $75-dollar electronic gift cards. A total of 6,484 (26%) participants—who met the inclusion criteria of being at least 18 years old and enrolled at the university at the time of study—completed the survey in 2019, and 12,536 (50%) participants completed the survey in 2020.

### Measures

Both surveys contained questions that provide key measures of food security, housing security, and potential determinants of these outcomes among survey respondents. To measure FS, authors used the validated survey questions and scoring procedures from the six-item short form of the U.S. Department of Agriculture (USDA) Household Food Security Survey Module (23, 24). The USDA survey questions ask about different aspects of household food security in the past 12 months, and each response option corresponds to a score. The responses to the six-item USDA survey were scored, summed, and categorized using the validated food security status groups reported in Bickel et al. (23). The resulting three categories of FS are: very low FS, low FS, and high or marginal FS. To measure HS, two survey questions were adapted—using input from college students in the target population—from the Los Angeles Community College District Survey of Student Basic Needs (25). The two HS measures were most suitable for the population of interest given the characteristics of their sample (25) and our community. The first HS survey question was: (Q18) “In the past 12 months, have you had a permanent address?” A “yes” response indicates higher HS, whereas a “no” response indicates lower HS. The second HS question was: (Q19) “Have you had to spend a night (or more) in any of the following: hotel or motel; home or room of a friend or acquaintance; home or room of a family member; shelter; transitional living center; public spaces like library, abandoned buildings, or a car.” Higher frequency responses indicate lower HS, whereas lower frequency responses indicate higher HS.

For measures of potential determinants of FS and HS, the survey asked questions on income, education (enrollment status and academic level), employment (status, location, and number of weekly h), age, gender, race/ethnicity, transportation (mode and reliability), and living situation. For the survey question on gender, respondents were asked to indicate their preferred pronouns (he/him, she/her, they/them, other, or prefer not to respond). Some of the standard questions were taken or adapted from the Los Angeles Community College District Survey of Student Basic Needs to meet our community characteristics (25). The study was IRB approved as exempt in September 2019 and amended and approved in 2020, and it was launched by the University's Dean of Students Office.

### Data cleaning and validation

**Figure 1:**
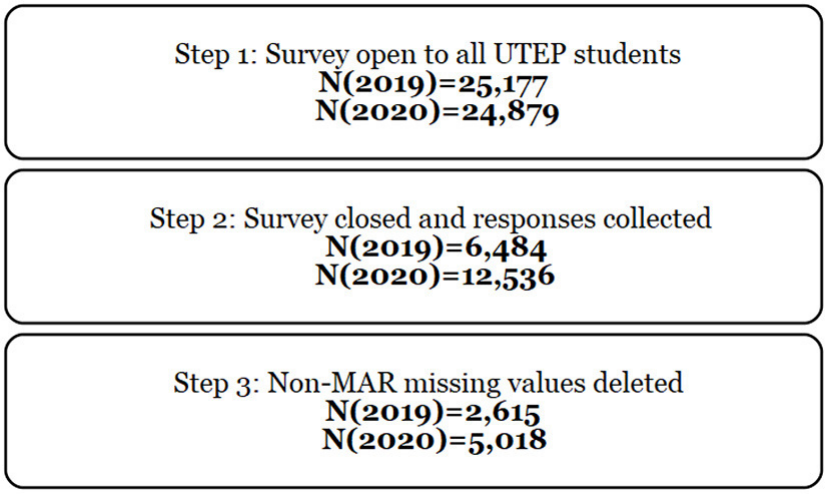
Consort diagram of 2019 and 2020 data collections.

All identifying information from the survey data was removed to protect confidentiality of participants, as well as responses with fully missing data. A missing value analysis was conducted for the remaining data in order to detect any further missing answers or patterns of missingness. However, data was deleted since missingness was not random (MAR) but exhibited strong patterns. Following this analysis, the observations that did not have levels recorded for food and housing security were deleted from the data. This results in a reduction in data as shown in Figure 1 consort diagram. Following this pre-processing stage, the data was readied for analysis by matching 28 variables common to both surveys. Some minor editing of variable levels was conducted in order to match results of the surveys. This was minor and inconsequential in each case except for household income where each year was aggregated to two levels (< $50,000 annual income and >=$50,000 annual income) since the levels provided as choices did not match with higher granularity. Finally, the USDA categories for food security were programmatically created using the six measures included in each year's survey. These categories were validated by the USDA (23) and are used for reporting out food security results.

### Statistical analysis

Descriptive statistics of the variables to both years were tested for association with the USDA food security outcomes and the housing security outcomes. When the factor was continuous, a simple F test from an ANOVA model was used to detect any difference in the means. When the data were categorical, exact Fisher tests with simulated *p*-values were used to test for association. These tests results were summarized with *p*-values in the analysis. Following the statistical tests, data visualizations were utilized to probe important factors that differ across the years. When a factor was deemed significant in 2020 but not 2019, we summarized this outcome using an appropriate visualization to understand the nature of the shift. All analyses were conducted in R (26) and made use of the ggplot2 (27) and summary (28) packages.

## Results

Initial analysis implies that food security increased from 2019 to 2020, and there is some evidence that housing security—as measured by a permanent address—increased as well (see Table 1). The housing security results are mixed, because a higher percentage of respondents reported (at least sometimes) experiencing a lack of any address in 2020. The housing and food security results are complex and due to a variety of factors, some of which may be temporary in nature. We explore the factors below, and we return to these findings in the discussion.

**Table 1:** Overall levels of food and housing security.

**Characteristic**	***N***	**2019**, ***N*** = **2,615**^a^	**2020**, ***N*** = **5,018**^a^
USDA rating	7,633		
Very Low FS		848 (32%)	1,174 (23%)
Low FS		618 (24%)	1,107 (22%)
High or marginal FS		1,149 (44%)	2,737 (55%)
(Missing)		0 (0%)	0 (0%)
Current living situation	7,627		
On campus		160 (6.1%)	131 (2.6%)
Off campus with family		1,832 (70%)	4,036 (80%)
Off campus no family		589 (23%)	804 (16%)
Other		28 (1.1%)	47 (0.9%)
Unknown		6	0
Permanent address	7,630		
Yes		2,331 (89%)	4,766 (95%)
No		281 (11%)	252 (5.0%)
Unknown		3	0
Frequency of no address	519		
Rarely		157 (59%)	136 (54%)
Sometimes		67 (25%)	83 (33%)
Often		43 (16%)	33 (13%)
Unknown		2,348	4,766

^a^*n (%)*.

**Figure 2:**
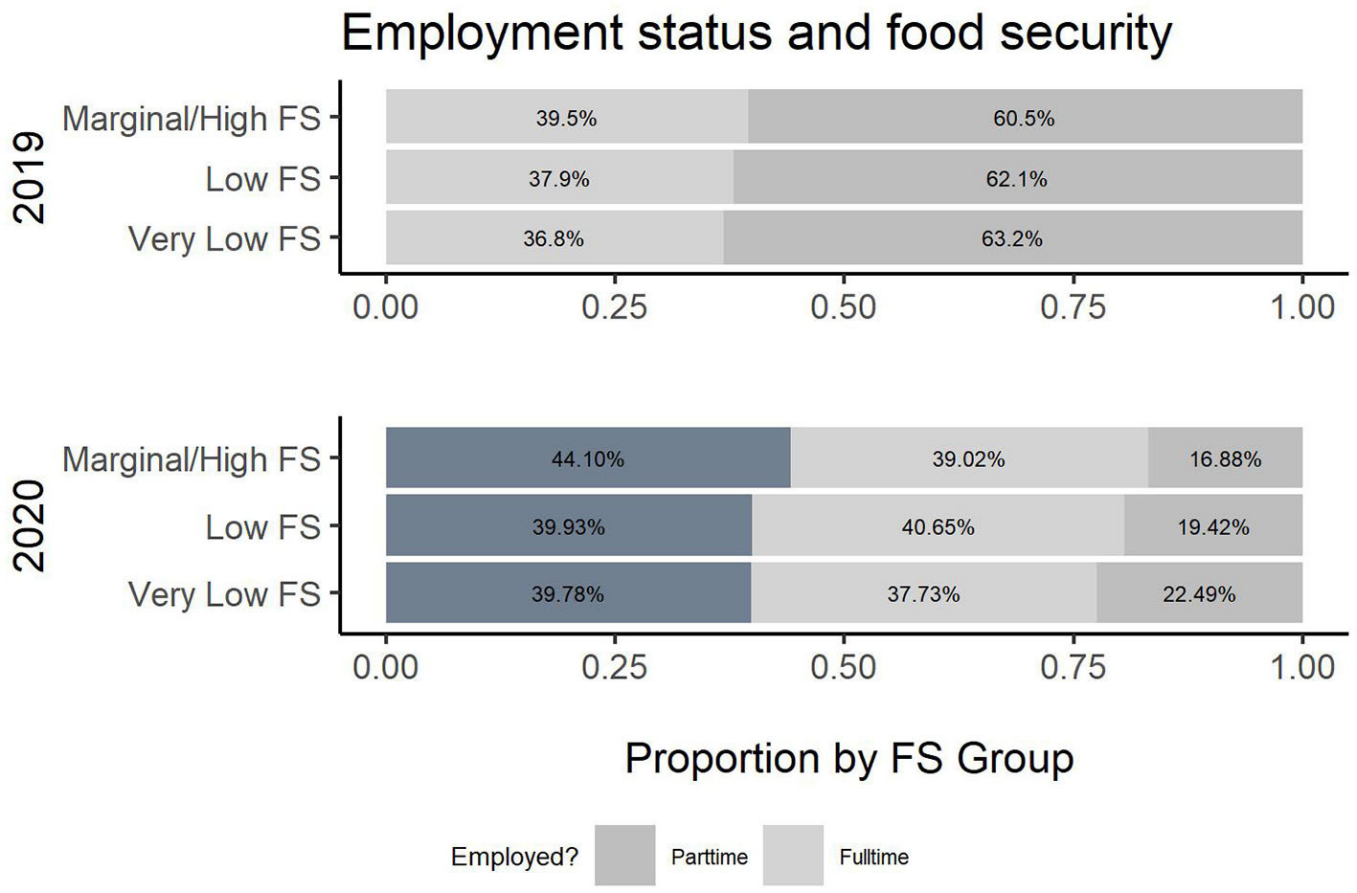
Employment status and food security.

**Figure 3:**
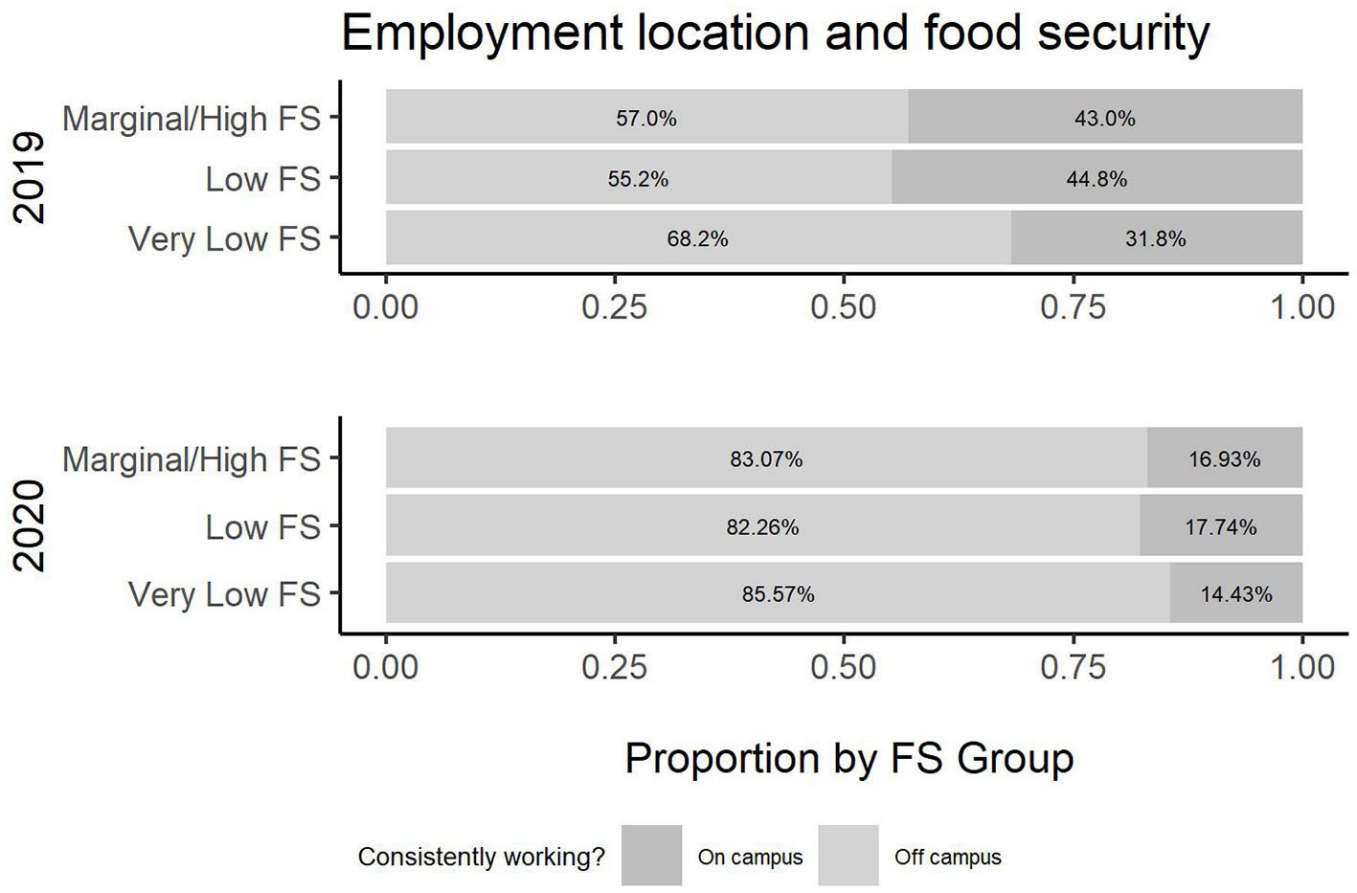
Employment location and food security.

**Figure 4:**
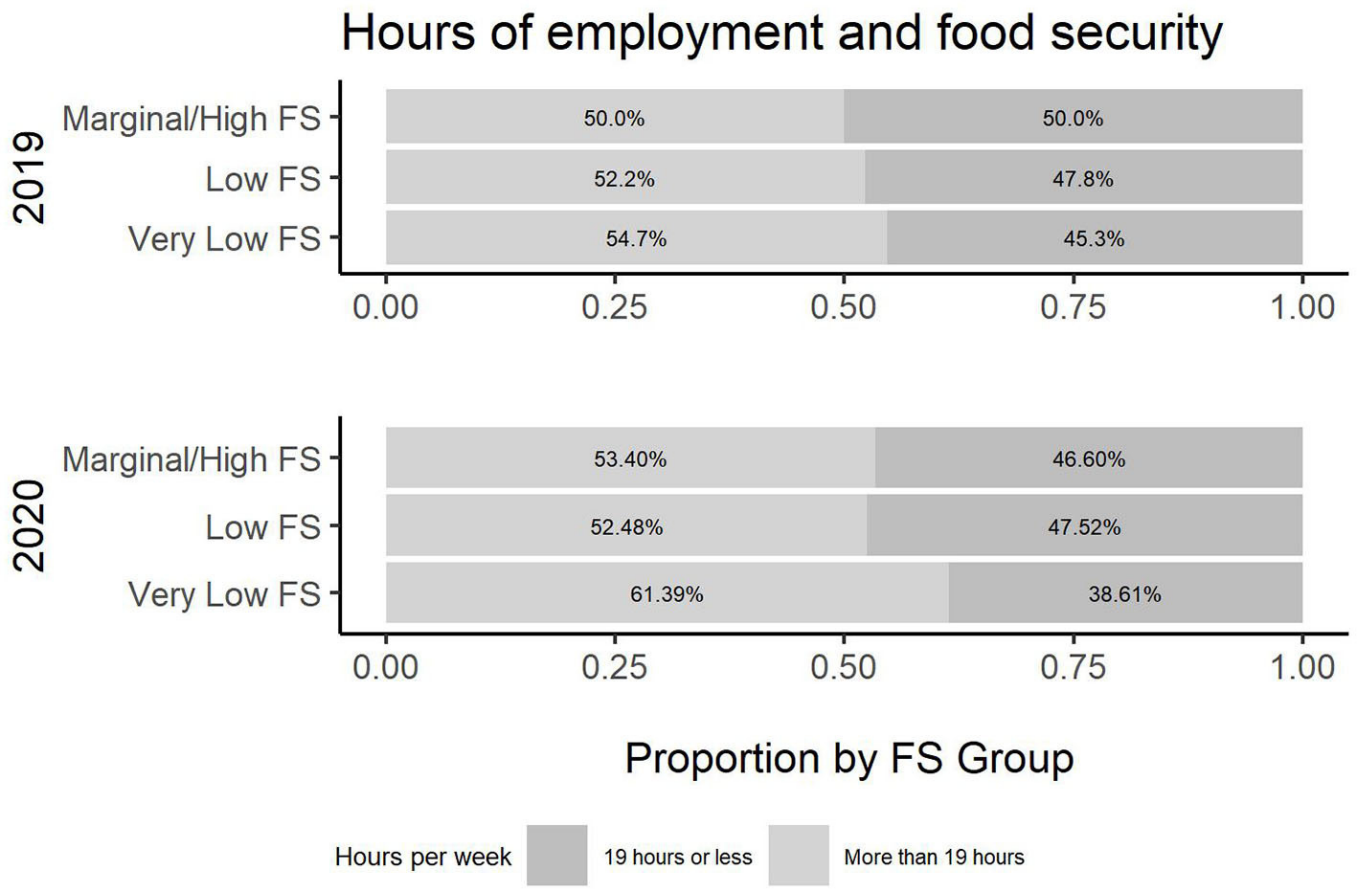
Hours worked per week and food security.

**Figure 5:**
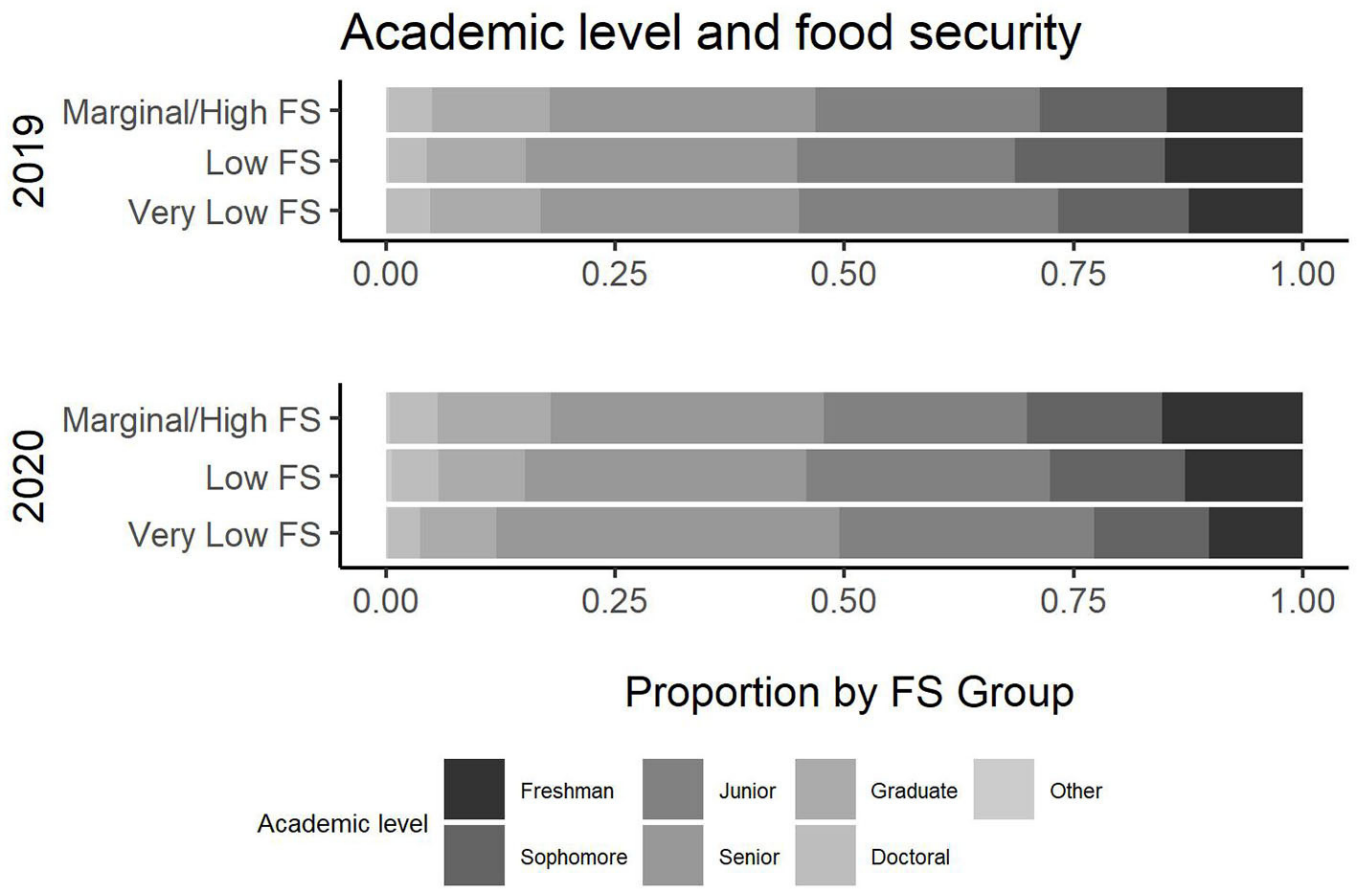
Academic level and food security.

**Figure 6:**
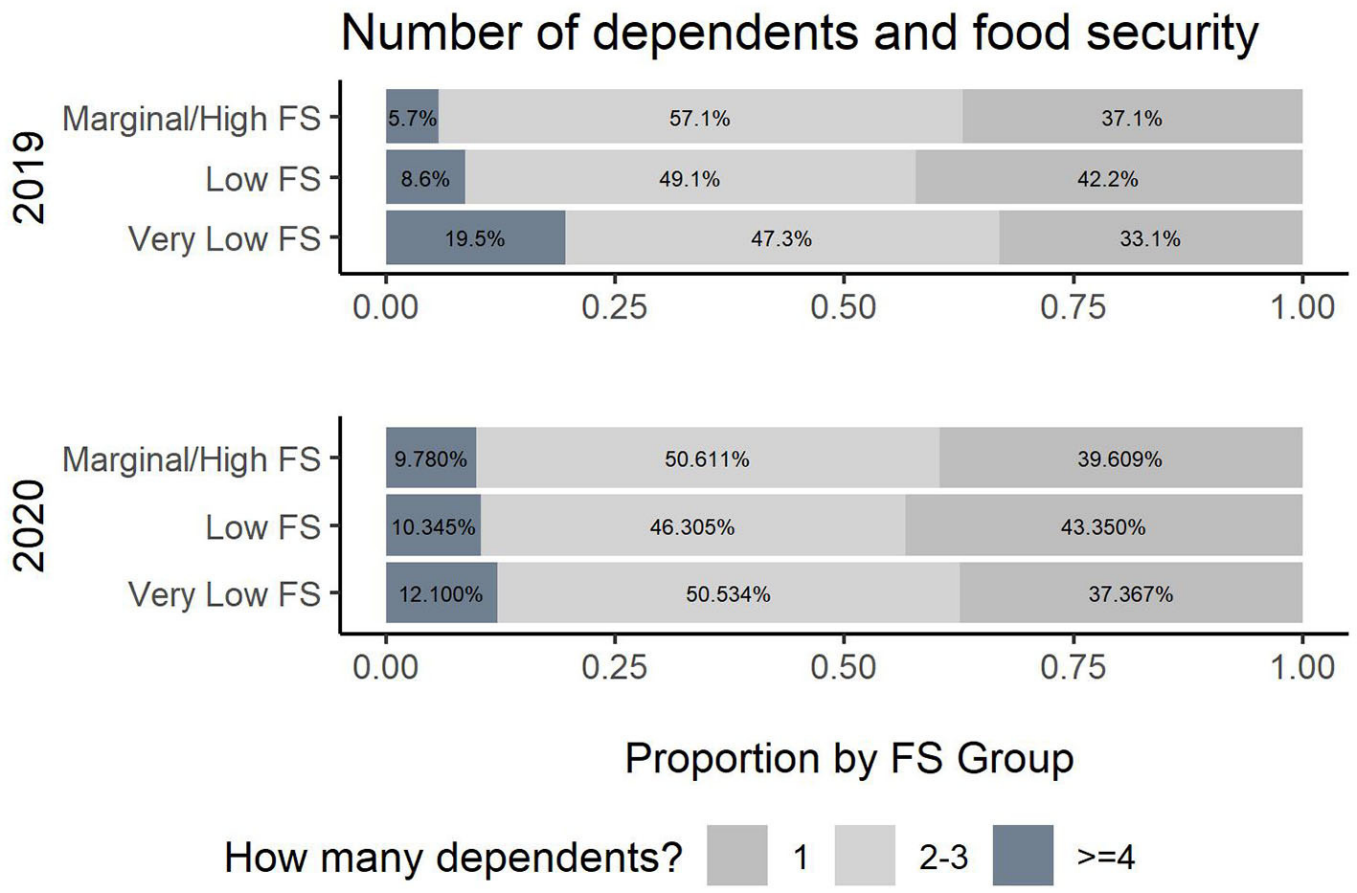
Number of dependents and food security.

**Figure 7:**
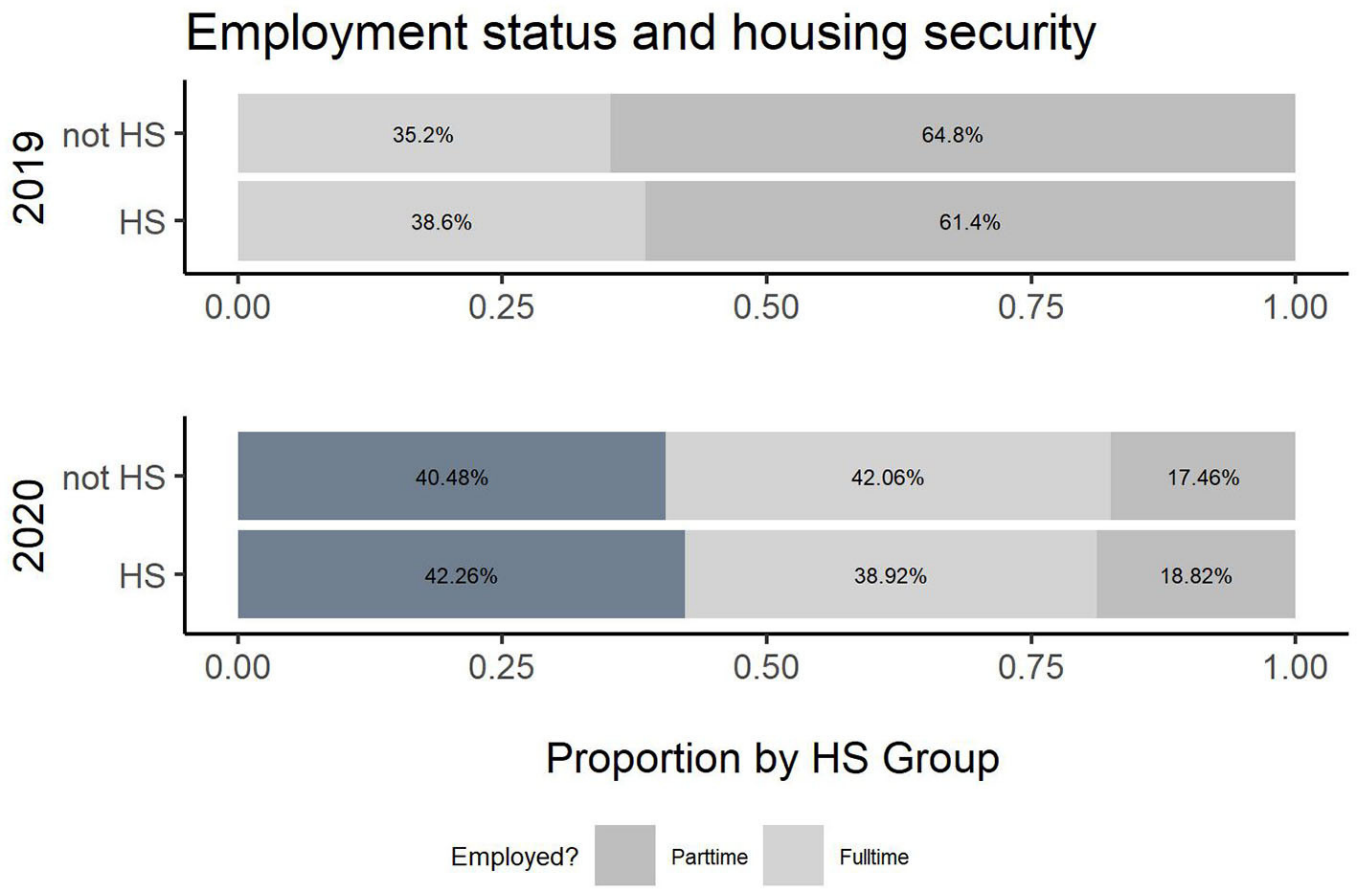
Employment status and housing security.

**Figure 8:**
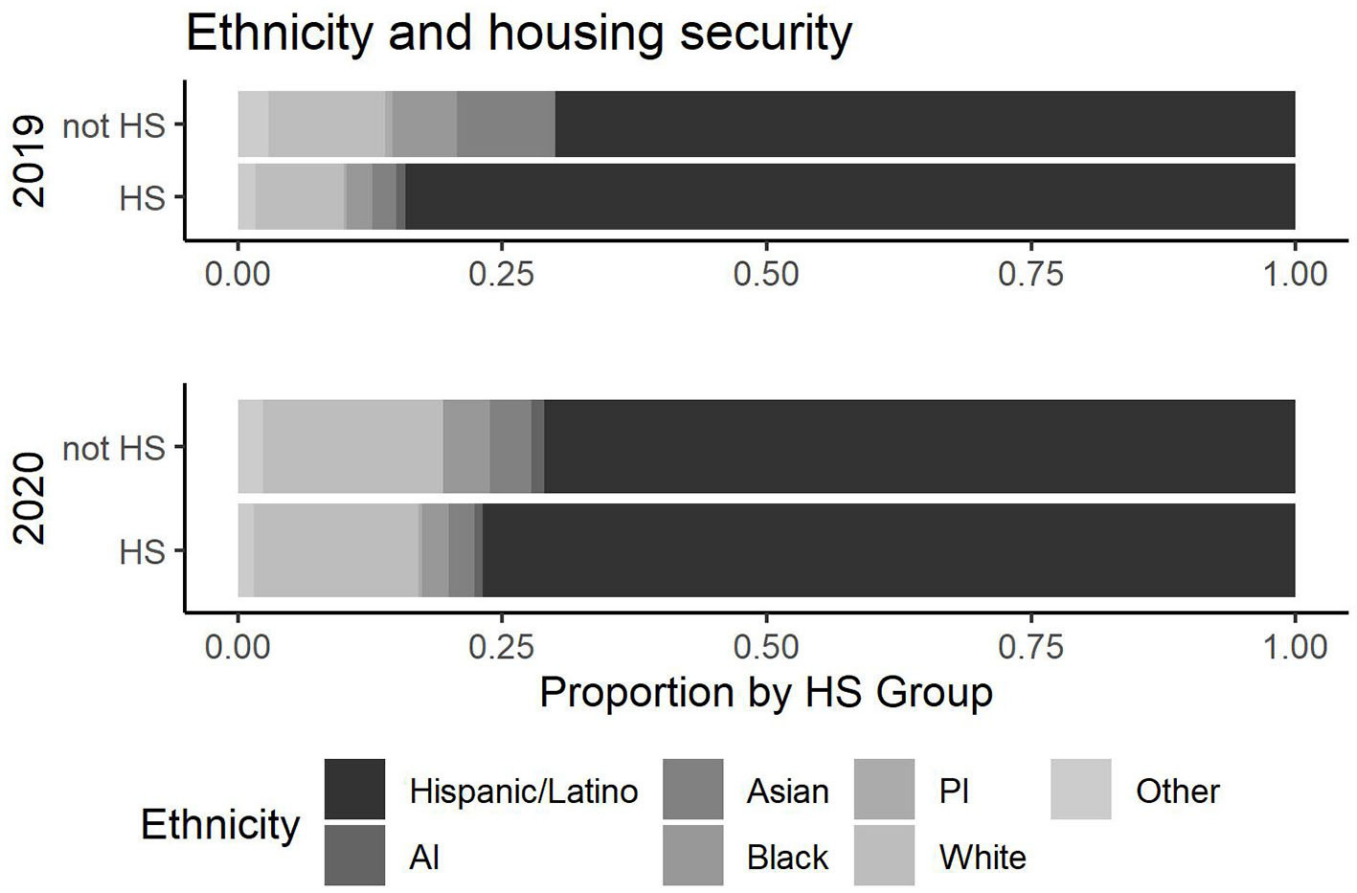
Ethnicity and housing security.

To investigate the intersectionality of food and housing security across 2019 and 2020 regarding gender, ethnicity, age, use of transportation, employment, being head of household, and income and public assistance, a more detailed table is produced. Table 2 presents the breakdown of all common variables across the years and by USDA food security category. Cochran-Mantel-Haenszel tests were performed for each variable and USDA rating stratified by Year. All tests were statistically significant, with the exception of Enrollment, demonstrating the need for the association analysis presented in Table 2. Additionally, Figures 2–8 illustrate the associations across the 2 years of the survey.

**Table 2:** Factors by year and USDA food insecurity group.

	**2019**				**2020**			
**Characteristic**	**Very Low FS**, ***N*** = **848**^a^	**Low FS**, ***N*** = **618**^*a*^	**High or Marginal FS**, ***N*** = **1,149**^a^	***p*** **-value** ^b^	**Very Low FS**, ***N*** = **1,174**^*a*^	**Low FS**, ***N*** = **1,107**^*a*^	**High or Marginal FS**, ***N*** = **2,737**^a^	***p*** **-value** ^b^
Enrollment				0.5				0.8
Full-time	727 (86%)	537 (87%)	974 (85%)		976 (83%)	932 (84%)	2,290 (84%)	
Part-time	119 (14%)	81 (13%)	175 (15%)		198 (17%)	175 (16%)	447 (16%)	
Employed?				0.4				<0.001
Full-time	536 (63%)	384 (62%)	695 (60%)		264 (22%)	215 (19%)	462 (17%)	
Part-time	312 (37%)	234 (38%)	454 (40%)		443 (38%)	450 (41%)	1,068 (39%)	
*N*o					467 (40%)	442 (40%)	1,207 (44%)	
Consistently working?				<0.001				0.2
On campus	170 (32%)	172 (45%)	298 (43%)		102 (14%)	118 (18%)	259 (17%)	
Off campus	365 (68%)	212 (55%)	395 (57%)		605 (86%)	547 (82%)	1,271 (83%)	
H per week				0.3				0.001
19 h or more	243 (45%)	183 (48%)	347 (50%)		273 (39%)	316 (48%)	713 (47%)	
Less than 19 h	293 (55%)	200 (52%)	347 (50%)		434 (61%)	349 (52%)	817 (53%)	
Age				<0.001				<0.001
<18	0 (0%)	1 (0.2%)	4 (0.3%)		5 (0.4%)	9 (0.8%)	15 (0.5%)	
19–24	548 (65%)	431 (70%)	843 (73%)		730 (62%)	757 (69%)	1,946 (71%)	
25–34	194 (23%)	132 (21%)	189 (16%)		295 (25%)	258 (23%)	510 (19%)	
35–44	71 (8.4%)	38 (6.2%)	79 (6.9%)		99 (8.4%)	54 (4.9%)	164 (6.0%)	
45–64	35 (4.1%)	15 (2.4%)	32 (2.8%)		43 (3.7%)	24 (2.2%)	98 (3.6%)	
>65	0 (0%)	0 (0%)	1 (<0.1%)		0 (0%)	1 (<0.1%)	2 (<0.1%)	
Family income				<0.001				<0.001
< $50,000	782 (93%)	526 (86%)	809 (71%)		993 (85%)	890 (80%)	1,711 (63%)	
>= $50,000	62 (7.3%)	88 (14%)	328 (29%)		181 (15%)	217 (20%)	1,026 (37%)	
Academic level				0.4				<0.001
Freshman	105 (12%)	93 (15%)	170 (15%)		120 (10%)	142 (13%)	421 (15%)	
Sophomore	121 (14%)	101 (16%)	159 (14%)		147 (13%)	163 (15%)	403 (15%)	
Junior	240 (28%)	147 (24%)	282 (25%)		327 (28%)	295 (27%)	607 (22%)	
Senior	239 (28%)	183 (30%)	333 (29%)		439 (37%)	339 (31%)	814 (30%)	
Masters	102 (12%)	67 (11%)	148 (13%)		98 (8.3%)	105 (9.5%)	339 (12%)	
Doctoral	41 (4.8%)	25 (4.0%)	54 (4.7%)		41 (3.5%)	57 (5.1%)	143 (5.2%)	
Professional	0 (0%)	2 (0.3%)	3 (0.3%)		2 (0.2%)	6 (0.5%)	10 (0.4%)	
Commute mode				<0.001				<0.001
Missing	29 (3.4%)	20 (3.3%)	46 (4.0%)		64 (5.5%)	44 (4.0%)	187 (6.8%)	
Car (alone)	502 (59%)	363 (59%)	756 (66%)		764 (65%)	719 (65%)	1,794 (66%)	
Carpool	83 (9.8%)	59 (9.6%)	115 (10%)		121 (10%)	129 (12%)	321 (12%)	
Bus/public	42 (5.0%)	33 (5.4%)	58 (5.1%)		52 (4.4%)	53 (4.8%)	100 (3.7%)	
Bike	103 (12%)	80 (13%)	102 (8.9%)		90 (7.7%)	100 (9.0%)	189 (6.9%)	
Trolley	11 (1.3%)	10 (1.6%)	6 (0.5%)		12 (1.0%)	6 (0.5%)	11 (0.4%)	
Walk	62 (7.3%)	39 (6.4%)	36 (3.1%)		49 (4.2%)	37 (3.3%)	56 (2.0%)	
Other	13 (1.5%)	10 (1.6%)	25 (2.2%)		21 (1.8%)	19 (1.7%)	78 (2.8%)	
Reliability of transportation				<0.001				<0.001
Not reliable	13 (1.5%)	3 (0.5%)	3 (0.3%)		40 (3.4%)	25 (2.3%)	47 (1.7%)	
Somewhat reliable	133 (16%)	46 (7.5%)	39 (3.4%)		192 (16%)	113 (10%)	134 (4.9%)	
Fairly reliable	320 (38%)	212 (35%)	283 (25%)		451 (38%)	464 (42%)	750 (27%)	
Very reliable	380 (45%)	352 (57%)	819 (72%)		491 (42%)	505 (46%)	1,806 (66%)	
Live alone?				<0.001				<0.001
Yes	134 (16%)	60 (9.7%)	73 (6.4%)		164 (14%)	104 (9.4%)	154 (5.6%)	
No	714 (84%)	556 (90%)	1,076 (94%)		1,010 (86%)	1,003 (91%)	2,583 (94%)	
Dependents?				<0.001				<0.001
Yes	169 (24%)	116 (21%)	175 (16%)		281 (28%)	203 (20%)	409 (16%)	
No	545 (76%)	439 (79%)	901 (84%)		729 (72%)	800 (80%)	2,174 (84%)	
How many?				0.002				0.6
1	56 (33%)	49 (42%)	65 (37%)		105 (37%)	88 (43%)	162 (40%)	
2–3	80 (47%)	57 (49%)	100 (57%)		142 (51%)	94 (46%)	207 (51%)	
>4	33 (20%)	10 (8.6%)	10 (5.7%)		34 (12%)	21 (10%)	40 (9.8%)	
Head of household				<0.001				<0.001
Yes	283 (33%)	158 (26%)	176 (15%)		404 (34%)	258 (23%)	464 (17%)	
No	565 (67%)	458 (74%)	970 (85%)		770 (66%)	849 (77%)	2,273 (83%)	
Current living situation				<0.001				<0.001
On campus	63 (7.4%)	39 (6.3%)	58 (5.1%)		58 (4.9%)	26 (2.3%)	47 (1.7%)	
Off campus with Family	503 (59%)	421 (68%)	908 (79%)		813 (69%)	876 (79%)	2,347 (86%)	
Off campus no family	267 (32%)	153 (25%)	169 (15%)		290 (25%)	194 (18%)	320 (12%)	
Other	14 (1.7%)	3 (0.5%)	11 (1.0%)		13 (1.1%)	11 (1.0%)	23 (0.8%)	
Permanent address				<0.001				<0.001
Yes	698 (82%)	555 (90%)	1,078 (94%)		1,065 (91%)	1,047 (95%)	2,654 (97%)	
No	150 (18%)	61 (9.9%)	70 (6.1%)		109 (9.3%)	60 (5.4%)	83 (3.0%)	
Frequency of no address				<0.001				<0.001
Rarely	65 (45%)	39 (66%)	53 (85%)		45 (41%)	30 (50%)	61 (73%)	
Somewhat	46 (32%)	15 (25%)	6 (9.7%)		49 (45%)	17 (28%)	17 (20%)	
Often	35 (24%)	5 (8.5%)	3 (4.8%)		15 (14%)	13 (22%)	5 (6.0%)	
Know of student homelessness				<0.001				<0.001
Yes	336 (40%)	166 (27%)	204 (18%)		417 (36%)	215 (19%)	326 (12%)	
No	511 (60%)	450 (73%)	945 (82%)		757 (64%)	892 (81%)	2,411 (88%)	
Ethnicity				0.002				<0.001
Hispanic/Latino	688 (81%)	522 (85%)	949 (83%)		881 (75%)	861 (78%)	2,101 (77%)	
American Indian	6 (0.7%)	5 (0.8%)	8 (0.7%)		17 (1.4%)	7 (0.6%)	14 (0.5%)	
Asian	17 (2.0%)	23 (3.7%)	40 (3.5%)		22 (1.9%)	34 (3.1%)	72 (2.6%)	
Black	31 (3.7%)	19 (3.1%)	23 (2.0%)		49 (4.2%)	35 (3.2%)	47 (1.7%)	
Pacific Islander	3 (0.4%)	2 (0.3%)	2 (0.2%)		5 (0.4%)	3 (0.3%)	9 (0.3%)	
White	81 (9.6%)	33 (5.3%)	112 (9.8%)		175 (15%)	151 (14%)	457 (17%)	
Other	21 (2.5%)	13 (2.1%)	13 (1.1%)		25 (2.1%)	16 (1.4%)	37 (1.4%)	
Gender (pronouns)				0.7				0.016
He/Him	265 (31%)	185 (30%)	330 (29%)		347 (30%)	314 (28%)	824 (30%)	
She/Her	568 (67%)	422 (68%)	806 (70%)		775 (66%)	752 (68%)	1,849 (68%)	
They/Them	6 (0.7%)	4 (0.6%)	4 (0.3%)		27 (2.3%)	18 (1.6%)	25 (0.9%)	
Other	8 (0.9%)	7 (1.1%)	9 (0.8%)		9 (0.8%)	5 (0.5%)	16 (0.6%)	
Prefer no answer					16 (1.4%)	18 (1.6%)	23 (0.8%)	
College				0.025				<0.001
Business administration	88 (10%)	56 (9.1%)	132 (11%)		136 (12%)	130 (12%)	293 (11%)	
Education	59 (7.0%)	49 (7.9%)	87 (7.6%)		100 (8.5%)	106 (9.6%)	261 (9.5%)	
Engineering	113 (13%)	112 (18%)	200 (17%)		156 (13%)	186 (17%)	473 (17%)	
Health sciences	109 (13%)	83 (13%)	174 (15%)		123 (10%)	127 (11%)	318 (12%)	
Liberal arts	266 (31%)	149 (24%)	280 (24%)		361 (31%)	262 (24%)	635 (23%)	
Science	155 (18%)	116 (19%)	199 (17%)		190 (16%)	173 (16%)	465 (17%)	
Nursing	45 (5.3%)	44 (7.1%)	61 (5.3%)		83 (7.1%)	96 (8.7%)	244 (8.9%)	
Pharmacy	6 (0.7%)	1 (0.2%)	7 (0.6%)		8 (0.7%)	16 (1.4%)	17 (0.6%)	
Other	7 (0.8%)	8 (1.3%)	9 (0.8%)		17 (1.4%)	11 (1.0%)	31 (1.1%)	

^a^*n (%)*.

^b^*Fisher's exact test for count data with simulated p-value (based on 2,000 replicates)*.

### Food security results

According to the survey results, several variables have a different relationship with food security across survey years. In Table 2, there is a change in the employment status across the 2019 and 2020 cohort and its association with food security (*p*-value (2019) =0.4, *p*-value (2020) <0.001). Figure 2 illustrates the change in employment status across the 2 years. Note that the level “no” was not an option in 2019 and, hence, excluded. Additionally, the location of employment differs in association across the years (*p*-value (2019) <0.001, *p-*value (2020) =0.2). Figure 3 illustrates this change in association. Finally, also regarding employment, the level of employment is also different across years (*p*-value (2019) =0.3, *p*-value (2020) <0.001), as demonatrated in Figure 4. In general, for the variables about employment status, there were more part-time employed students and fewer students working on campus during the pandemic than before. Moreover, the association between this and being food secure was associated with employment variables.

Regarding variables focused on student characteristics, there was an association now between academic level and food security that did not exist prior to the pandemic (see Figure 5). More senior and junior students were having issues with food security relative to other academic levels. The number of dependents also was no longer associated with food security (*p-*value (2019) =0.002, *p*-value (2020) =0.6). This was indicated particularly by less impact by number of dependents. Finally, other students' characteristics were associated with food security across both data collections.

### Housing security results

The survey results also demonstrate changes in relationships between some key variables and housing security across survey years. Table 3 presents the results on housing security and factors associated. As with Table 2, Cochran-Mantel-Haenszel tests were performed for each variable and hunger status with Year as the stratification variable. Again, all tests were statistically significant, with the exception of Enrollment. Regarding housing security (permanent housing-yes or no), there was a slight difference in association for employment status and housing security (*p*-value (2019) = 0.03, *p*-value (2020) = 0.08). This indicates that more full-time students were housing secure during the pandemic as depicted in Figure 7.

**Table 3:** Factors by year and housing security group.

	**2019**			**2020**		
**Characteristic**	**Yes**, ***N*** = **2,331**^a^	**No**, ***N*** = **281**^*a*^	***p*** **-value** ^b^	**Yes**, ***N*** = **4,766**^a^	**No**, ***N*** = **252**^a^	***p*** **-value** ^b^
Enrollment			0.030			0.080
Full-time	1,983 (85%)	252 (90%)		3,977 (83%)	221 (88%)	
Part-time	347 (15%)	28 (10%)		789 (17%)	31 (12%)	
Employed?			0.3			0.6
Full-time	1,432 (61%)	182 (65%)		897 (19%)	44 (17%)	
Part-time	899 (39%)	99 (35%)		1,855 (39%)	106 (42%)	
No				2,014 (42%)	102 (40%)	
Consistently working?			0.6			0.11
On campus	563 (39%)	76 (42%)		447 (16%)	32 (21%)	
Off campus	866 (61%)	106 (58%)		2,305 (84%)	118 (79%)	
Hours per week			0.4			0.9
19 h or more	691 (48%)	82 (45%)		1,236 (45%)	66 (44%)	
Less than 19 h	739 (52%)	100 (55%)		1,516 (55%)	84 (56%)	
Age			0.003			0.014
<18	4 (0.2%)	1 (0.4%)		28 (0.6%)	1 (0.4%)	
19–24	1,633 (70%)	187 (67%)		3,244 (68%)	189 (75%)	
25–34	438 (19%)	76 (27%)		1,010 (21%)	53 (21%)	
35–44	179 (7.7%)	9 (3.2%)		310 (6.5%)	7 (2.8%)	
45–64	74 (3.2%)	8 (2.8%)		163 (3.4%)	2 (0.8%)	
>65	1 (<0.1%)	0 (0%)		3 (<0.1%)	0 (0%)	
Family income			<0.001			<0.001
< $50,000	1,854 (80%)	261 (94%)		3,373 (71%)	221 (88%)	
>= $50,000	461 (20%)	17 (6.1%)		1,393 (29%)	31 (12%)	
Academic level			0.10			0.3
Freshman	328 (14%)	40 (14%)		642 (13%)	41 (16%)	
Sophomore	339 (15%)	42 (15%)		674 (14%)	39 (15%)	
Junior	596 (26%)	71 (25%)		1,163 (24%)	66 (26%)	
Senior	689 (30%)	66 (23%)		1,527 (32%)	65 (26%)	
Masters	275 (12%)	41 (15%)		517 (11%)	25 (9.9%)	
Doctoral	99 (4.2%)	21 (7.5%)		225 (4.7%)	16 (6.3%)	
Professional	5 (0.2%)	0 (0%)		18 (0.4%)	0 (0%)	
Commute mode			<0.001			<0.001
Missing	79 (3.4%)	16 (5.7%)		284 (6.0%)	11 (4.4%)	
Car (alone)	1,484 (64%)	136 (49%)		3,127 (66%)	150 (60%)	
Carpool	234 (10%)	22 (7.9%)		546 (11%)	25 (9.9%)	
Bus/public	124 (5.3%)	9 (3.2%)		195 (4.1%)	10 (4.0%)	
Bike	237 (10%)	48 (17%)		363 (7.6%)	16 (6.3%)	
Trolley	19 (0.8%)	8 (2.9%)		27 (0.6%)	2 (0.8%)	
Walk	102 (4.4%)	35 (12%)		116 (2.4%)	26 (10%)	
Other	42 (1.8%)	6 (2.1%)		107 (2.2%)	11 (4.4%)	
Reliability of transportation			0.003			<0.001
Not reliable	18 (0.8%)	1 (0.4%)		103 (2.2%)	9 (3.6%)	
Somewhat reliable	185 (8.0%)	33 (12%)		397 (8.3%)	42 (17%)	
Fairly reliable	709 (31%)	106 (38%)		1,563 (33%)	102 (40%)	
Very reliable	1,408 (61%)	141 (50%)		2,703 (57%)	99 (39%)	
Live alone?			<0.001			<0.001
Yes	204 (8.8%)	63 (22%)		361 (7.6%)	61 (24%)	
No	2,126 (91%)	218 (78%)		4,405 (92%)	191 (76%)	
Dependents?			>0.9			>0.9
Yes	417 (20%)	42 (19%)		857 (19%)	36 (19%)	
No	1,708 (80%)	176 (81%)		3,548 (81%)	155 (81%)	
How many?			0.015			0.036
1	147 (35%)	23 (55%)		336 (39%)	19 (53%)	
2–3	218 (52%)	18 (43%)		426 (50%)	17 (47%)	
4 or more	52 (12%)	1 (2.4%)		95 (11%)	0 (0%)	
Head of household			<0.001			<0.001
Yes	501 (22%)	115 (41%)		1,029 (22%)	97 (38%)	
No	1,826 (78%)	166 (59%)		3,737 (78%)	155 (62%)	
Current living situation			<0.001			<0.001
On campus	117 (5.0%)	43 (15%)		113 (2.4%)	18 (7.1%)	
Off campus with family	1,744 (75%)	86 (31%)		3,920 (82%)	116 (46%)	
Off campus no family	444 (19%)	145 (52%)		690 (14%)	114 (45%)	
Other	21 (0.9%)	7 (2.5%)		43 (0.9%)	4 (1.6%)	
Know of student homelessness			0.6			0.011
Yes	626 (27%)	80 (29%)		894 (19%)	64 (25%)	
No	1,704 (73%)	200 (71%)		3,872 (81%)	188 (75%)	
USDA rating			<0.001			<0.001
Very low FS	698 (30%)	150 (53%)		1,065 (22%)	109 (43%)	
Low FS	555 (24%)	61 (22%)		1,047 (22%)	60 (24%)	
High or marginal FS	1,078 (46%)	70 (25%)		2,654 (56%)	83 (33%)	
(Missing)	0 (0%)	0 (0%)		0 (0%)	0 (0%)	
Ethnicity			<0.001			0.13
Hispanic/Latino	1,960 (84%)	196 (70%)		3,664 (77%)	179 (71%)	
AI	19 (0.8%)	0 (0%)		35 (0.7%)	3 (1.2%)	
Asian	54 (2.3%)	26 (9.3%)		118 (2.5%)	10 (4.0%)	
Black	56 (2.4%)	17 (6.1%)		120 (2.5%)	11 (4.4%)	
PI	5 (0.2%)	2 (0.7%)		17 (0.4%)	0 (0%)	
White	195 (8.4%)	31 (11%)		740 (16%)	43 (17%)	
Other	39 (1.7%)	8 (2.9%)		72 (1.5%)	6 (2.4%)	
Gender (pronouns)			0.011			0.090
He/Him	682 (29%)	97 (35%)		1,412 (30%)	73 (29%)	
She/Her	1,617 (69%)	177 (63%)		3,210 (67%)	166 (66%)	
They/Them	9 (0.4%)	5 (1.8%)		66 (1.4%)	4 (1.6%)	
Other	22 (0.9%)	2 (0.7%)		25 (0.5%)	5 (2.0%)	
Prefer no answer				53 (1.1%)	4 (1.6%)	
College			0.022			0.023
Business administration	250 (11%)	26 (9.3%)		527 (11%)	32 (13%)	
Education	179 (7.7%)	16 (5.7%)		457 (9.6%)	10 (4.0%)	
Engineering	366 (16%)	58 (21%)		784 (16%)	31 (12%)	
Health sciences	332 (14%)	32 (11%)		536 (11%)	32 (13%)	
Liberal arts	607 (26%)	88 (31%)		1,188 (25%)	70 (28%)	
Science	418 (18%)	52 (19%)		776 (16%)	52 (21%)	
Nursing	143 (6.1%)	7 (2.5%)		403 (8.5%)	20 (7.9%)	
Pharmacy	14 (0.6%)	0 (0%)		38 (0.8%)	3 (1.2%)	
Other	22 (0.9%)	2 (0.7%)		57 (1.2%)	2 (0.8%)	

^a^*n (%)*.

^b^*Fisher's exact test for count data; Fisher's exact test for count data with simulated p-value (Based on 2,000 replicates)*.

Ethnicity also indicates a decrease in Hispanic/Latino students during the pandemic who have permanent housing as shown in Figure 8 (*p*-value (2019) <0.001, *p*-value (2020) =0.13). Other variables were and remain to be associated with housing security across 2019 and 2020.

## Discussion

The results suggest that food security and one dimension of housing security—possessing a permanent address—improved among university students in the 2019 and 2020 samples. Specifically, levels of high or marginal food security increased from 44 in 2019 to 55% in 2020; levels of very low food security decreased from 32 in 2019 to 23% in 2020; and possessing a permanent address increased from 89 in 2019 to 95% in 2020. In contrast, for the second measure of housing security (the frequency of lacking any address), there was an increase in the percentage of students who reported that at least sometimes they lacked any address.

Despite the pandemic's upheaval of academic, economic, and social structures, our findings demonstrate that fewer students at this HSI experienced very low food security and (one form of) low housing security during the first year of the pandemic. We are unable to determine why food and housing security improved among university students during the pandemic, but social assistance interventions—including the expanded efforts by the government, community organizations, and the University—may have played a key role (29–31). It also is important to note that the percentage of students in the sample who lived off campus with family increased from 70 in 2019 to 80% in 2020 (see Table 1), which could account for some of the increase in food security. Below we highlight some key factors that are associated with student food and housing security across years and subgroups, and in the next subsection we describe the University's efforts and develop a new model to improve food and housing security. University services prior and during the COVID-19 pandemic are listed in Table 4.

**Table 4:** University model to address food and housing insecurity.

**Pre-COVID pandemic food and housing support services**	**Changes to the food and housing support services influenced by COVID**
University Food Pantry established in 2014, operated first out of a modest closet, and expanded in 2018 to an office inside a gymnasium facility and across from student dormitories with convenient parking to support students.	The magnitude of FI and HI among students in 2019 and 2020, along with the associations across years, were influenced by the efforts of the University.
Efforts centered on providing emergency food assistance *via* pantry and emergency support for foster students and students experiencing homelessness. Food pantry referred students to the local food bank, pantries and health and human service organizations. Pantry offerings consisted of packaged grains, cereals, fruit, tuna, chicken, and toiletries (32, 33).	University shifted to provide a range of financial assistance and support services. Pantry was one of the few sites that remained operational due to the essential service it provided. Campus pantry adapted its model to seek donations through social media and a digital platform, where donors could browse, purchase and send non-perishable items delivered directly to campus. Additional investments in the pantry by the University to help meet growing student needs and expanded its efforts by providing grocery store gift cards and donating additional holiday gift baskets to ensure that students had sufficient food during long holidays (32).
In addition, the Foster, Homeless, and Adopted Resources (FHAR) Program provided financial and other support services for students with severe housing insecurity (33).	University used federal COVID Relief funds to provide housing grants for on-campus housing expenses. Opened dormitories for emergency housing and offered support services to connect students to more permanent housing off campus. Increased investments in the FHAR Program (33).
	Introduced diverse emergency financial assistance to serve as safety net to pay for food and rent. Raised private contributions to create emergency aid fund. Over $71 million of federal funds were for tuition grants. Short-term emergency loans to assist with basic needs (34, 35).
	Increased awareness of resources available and encouraged use. Faculty shared resources with students in class, syllabus, and encouraged them to utilize resources. Counseling and psychological services expanded services and shifted to a combination of in-person and telehealth services (36).

Employment status and other related employment variables were altered during the pandemic. Nationally, many who had worked full-time reduced their employment to part-time status or no employment (37). This change in employment status, along with a halting on payment plans for student loans and the financial assistance provided by the CARES Act (38), may have affected the changes in association with food and housing security. The results suggest that educational and higher education institutions need to shift to providing more employment opportunities to students on campus when possible and consider that many students are still struggling to adjust to the end of CARES funding and will need additional income generating opportunities.

It is important to emphasize that the student population at an HSI is not monolithic: key differences in food and housing security exist across subgroups. For example, regarding housing security, it is evident that Hispanic students experienced a decreased access to permanent housing. Pre-pandemic, 84% of Hispanic students had access to permanent housing and during the pandemic it decreased to 77%. This presents an opportunity for higher education and educational institutions to address this change by providing support services centered on locating affordable housing on and off campus. Considering this evidence, it is recommended that educational institutions be flexible and responsive regarding needs for affordable and accessible housing, and University leaders may want to target information campaigns to vulnerable student groups.

Overall, the article has some important strengths. Food and housing security is assessed among students at an HSI. Previous studies often have low percentages of Hispanic students, so the results fill a key gap in our understanding of food and housing security in higher education. In addition, the article presents food and housing security data both before and during the pandemic. By assessing food and housing security in two different time periods, the article improves our understanding of how food and housing security changed after the start of the pandemic. Furthermore, the study has high survey response rates. The high response rates by students may be due to the use of a trusted online survey platform and convenient email distribution methods.

### Recommendations

Along with other forms of social assistance, University interventions can play an important role in addressing basic needs and inequities among HSI higher education students. Given the bio-psycho-social-economic factors and stressors associated with the COVID-19 pandemic, it is imperative to provide students with continued financial, psychological and support services to mitigate the medium- and long-term effects of the pandemic. Government tuition and relief support programs are needed to help students in their education, to provide nutrition and housing to struggling students, and to improve the quality of life of the community.

Tailored interventions are needed (1) to address stigma associated with accessing psychological, counseling, food and housing support services, and (2) to meet student's cultural and linguistic realities. To assist with student retention and academic success, it is key to reduce barriers, such as chronic hunger and sustained risk of unstable housing. Food distribution centers on campus are key environments to assist students in acquiring enough nutrient-dense food to overcome dietary limitations and reduce health disparities. It is important to orient students on public assistance and other campus and community resources to increase FS and HS, including the existence and eligibility of the Supplemental Nutrition Assistance Program (SNAP); Special Supplemental Nutrition for Women, Infants, and Children (WIC); Medicaid; Children's Health Insurance Program (CHIP); and local food banks and hunger relief centers. In the informational campaigns, a special emphasis should be placed on reaching vulnerable student subgroups, including those who work, are head of household, have children, receive health and human services, and have limited or no transportation. Instructors can provide information on assistance resources in the course syllabus, program/department web pages and social media pages. The establishment and promotion of campus-based programs and services through no-questions-asked food distribution and assistance venues for students is necessary. It also is essential to develop and implement food, housing and financial security tools for higher education students, so that the University can provide programming on campus to promote a secure campus environment with visual appeal, a comprehensive safety net, and culturally and linguistically responsive services (36).

Based on the study results and the reviewed literature, we conclude that it is important to bring access and excellence to pantry models of emergency food assistance. For this reason, we propose a new model, where the academy works across disciplines and implements policies to increase access, mitigate stigma, ensure nutritional education and launch integrated eligibility for public assistance and other valuable support services for students. These innovations will provide students with needed protections from food and housing insecurity, advance discovery of public value, and positively impact the education, economy, health, and culture of the community. A proposed model to improve food and housing security on campus is found in Table 5.

**Table 5:** Call to action.

Ensure that nutritious food options are activated and utilized	Generate a meal-sharing program, in which students, faculty or staff can donate food credits or swipes.
	Pantries with perishable, frozen and non-perishable items of high nutritional value, with online and pick up options.
	Open an integrated eligibility office to enroll in SNAP and other public benefits.
	Offer nutrition and health promotion education through professionals to orient on nutrients and meal preparation.
	Collaborate up with campus food services, food banks, and community-based organizations to bring hot meal kitchen services to campus.
Inform of external food distribution centers and housing assistance sites	Generate and disseminate directories of housing, food, transportation, health and human services online and hard copies.
	Identify and participate in health fairs and community events to promote food and housing security. Post event announcements on the online and bulletin boards, campus venues and student health centers.
Reduce stigma surrounding use support services	Ensure that course syllabus includes resource links to food, housing, transportation and other support services and encourage faculty to promote access.
	Offer regular tours to faculty, staff and student advisors of the university food pantry and Foster Homeless and Adopted Resources and promote access.
	Motivate faculty, staff and students to visit the support services on campus to demystify and mitigate stigma.
	Secure grants, financial or in-kind support from private and public donors and funders to increase the food bank's nutritious options and make campus food services affordable to students.
	Rename campus food pantry based on student input to make to more inclusive.
	Conduct ongoing food and housing security assessments to inform campus leadership on way to address social and political determinants.
Create opportunities for community-engaged scholarship	Engage faculty, staff and students in the development and implementation of a food and housing security strategy.
	Designate student ambassadors or advisors in Campus Colleges and Schools to promote food, housing and transportation security.
Institutionalize support services	Generate policies to secure and expand nutritional food services and improve access to affordable housing, transportation, and health services.
	Develop a food, housing and financial security toolkit to guide programming on campus.
	Ensure adequate space, equipment, and personnel for food storage and distribution.
	Include the food pantry and student support services in university interactive maps and expand h of operation evenings and weekends to meet the needs of working students.

### Study limitations

The study contains some key limitations. The cross-sectional study design limits our ability to make causal inferences regarding key factors and food and housing security. Also, the self-reported instrument relies primarily on subjective responses from students, which may be biased. Furthermore, food- and housing-insecure students may be less likely to respond to a survey, which will overestimate food and housing security levels. Despite these limitations, the findings from this study have several important implications for research, practice and policy.

## Conclusion

The current study contributes to the literature on food and housing security in higher education by focusing on college students—both before and during a pandemic—at an HSI. Higher education plays an important role in the generation of social capital, mobility, and health. To ensure that university students thrive academically, succeed socially and ultimately graduate, it is necessary to ensure that education institutions secure food and housing assistance for marginalized and vulnerable populations.

Designing programs and policies with input from students is essential if we want to increase the utilization of assistance and prevent hunger and homelessness. Being responsive to changes in food or housing security also is crucial and requires concerted work to achieve. Multidisciplinary and collaborative work is required to mitigate food insecurity on campus, advance health and academic outcomes, improve the on-campus food and housing environments, and provide subsidized food options to facilitate equitable access to food. These efforts require guidance from health professionals, including nutritionists to assist students with meal preparation and budgeting skills. Ensuring equitable access to healthy food and affordable housing on campus is essential. Future research can evaluate the use and effectiveness of campus resources in improving food and housing security of university students.

The challenges of the pandemic create an opportunity for universities to strengthen food and housing security among students. Economic and health crises do not guarantee increased levels of basic needs insecurity. Instead, higher education institutions can shift to a new, more comprehensive model of food and housing assistance. The model shift will improve student basic needs security and academic outcomes, increase opportunities for higher education and upward social mobility, and create stronger and more successful communities.

## Data availability statement

The original contributions presented in the study are included in the article/supplementary material, further inquiries can be directed to the corresponding author/s.

## Ethics statement

The studies involving human participants were reviewed and approved by University of Texas at El Paso (IRB number 1470143). The patients/participants provided their written informed consent to participate in this study.

## Author contributions

Conceptualization, writing—review and editing, and writing—original draft preparation: EM, AW, GS, and SC-B. Methodology: EM, AW, and GS. Analysis: AW. Investigation: EM, GS, and JA. Visualization: AW and PD. Project administration: EM and JA. All authors contributed to the article and approved the submitted version.

## Funding

This publication was supported by the National Institute on Minority Health and Health Disparities of the National Institutes of Health under Award Number U54MD007592. The content is solely the responsibility of the authors and does not necessarily represent the official views of the National Institutes of Health.

## Conflict of interest

The authors declare that the research was conducted in the absence of any commercial or financial relationships that could be construed as a potential conflict of interest.

## Publisher's note

All claims expressed in this article are solely those of the authors and do not necessarily represent those of their affiliated organizations, or those of the publisher, the editors and the reviewers. Any product that may be evaluated in this article, or claim that may be made by its manufacturer, is not guaranteed or endorsed by the publisher.
